# Supplementary material for: Brain network changes after the first seizure: an insight into medication response?
Source: Brain Commun. 2024 Sep 20;6(5):fcae328. doi: 10.1093/braincomms/fcae328 (PMC11495098; doi:10.1093/braincomms/fcae328)
Supplement: fcae328_Supplementary_Data [file fcae328_supplementary_data.docx]

**Supplementary Materials 1:**

A T1-weighted (T1w) T1w image (TR = 1900 s, TE = 2500 ms and 0.9x0.9x0.9 mm voxel size) and two 15-minute long resting-state fMRI scans (pre-ASM and post-ASM) were completed for each participant (TR = 3000 ms, TE = 30 ms and 3x3x3 mm voxel size). Data preprocessing was performed using fMRIPrep ^20,^ , which is based on Nipype ^2^. T1w images were corrected for intensity non-uniformity with N4BiasFieldCorrection ^3^ in ANTs ^4^. The T1w-reference was then skull-stripped with a Nipype implementation of the antsBrainExtraction.sh workflow (from ANTs), using OASIS30ANTs as the target template. Brain tissue segmentation of cerebrospinal fluid, white matter and grey matter was performed on the brain-extracted T1w using fast in FSL (the FMRIB Software Library) ^5^. An anatomical T1w-reference map was computed after registration of the T1w images (after intensity non-uniformity correction) using mri_robust_template in FreeSurfer ^6^. Brain surfaces were also reconstructed using recon-all in FreeSurfer ^7^, and the brain mask estimated previously was refined with a custom variation of the method to reconcile ANTs-derived and FreeSurfer-derived segmentations of the cortical grey matter of Mindboggle ^8^. Volume-based spatial normalization to standard spaces (MNI152NLin6Asym, MNI152NLin2009cAsym) was performed through nonlinear registration with antsRegistration, using brain-extracted versions of both T1w reference and the T1w template. A deformation field to correct susceptibility distortions was estimated based on fMRIPrep's fieldmap-less approach. The deformation field results from co-registering the fMRI reference to the same-subject T1w-reference with its intensity inverted ^9,10^. Registration was performed with antsRegistration, and the process is regularized by constraining deformation to be nonzero only along the phase-encoding direction and modulated with an average field map template ^11^.

The following preprocessing was performed for each fMRI session (pre- and post-ASM). First, a reference volume and its skull-stripped version were generated using a custom methodology of fMRIPrep. Head-motion parameters for the fMRI reference (transformation matrices and six corresponding rotation and translation parameters) are estimated before spatiotemporal filtering using mcflirt in FSL ^12^. The calculated field map was aligned with rigid registration to the target echo-planar imaging reference run. The field coefficients were mapped onto the reference echo-planar imaging using the transform. The fMRI reference was then co-registered to the T1w reference using bbregister (FreeSurfer), which implements boundary-based registration ^13^. Co-registration was configured with six degrees of freedom. The signals are extracted within the cerebrospinal fluid, the white matter, and the whole-brain masks (i.e., the global signal). The fMRI time series were resampled into standard space, generating a preprocessed fMRI run in MNI152NLin6Asym space. First, a reference volume and its skull-stripped version were generated using a custom methodology of fMRIPrep. All resamplings can be performed with a single interpolation step by composing all the pertinent transformations (i.e. head-motion transform matrices, susceptibility distortion correction when available, and co-registrations to anatomical and output spaces). Gridded (volumetric) resamplings were performed using antsApplyTransforms (ANTs), configured with Lanczos interpolation to minimize the smoothing effects of other kernels ^14^. Non-gridded (surface) resamplings were performed using mri_vol2surf in FreeSurfer.

**References:**

1. Esteban O, Markiewicz CJ, Blair RW, Moodie CA, Isik AI, Erramuzpe A, et al. fMRIPrep: a robust preprocessing pipeline for functional MRI. Nature Methods. 2019; 16(1):111–6.

2. Gorgolewski K, Burns CD, Madison C, Clark D, Halchenko YO, Waskom ML, et al. Nipype: A Flexible, Lightweight and Extensible Neuroimaging Data Processing Framework in Python. Front Neuroinform [Internet]. 2011 [cited 2020]; 5. Available from: https://www.frontiersin.org/articles/10.3389/fninf.2011.00013/full

3. Tustison NJ, Avants BB, Cook PA, Zheng Y, Egan A, Yushkevich PA, et al. N4ITK: Improved N3 Bias Correction. IEEE Transactions on Medical Imaging. 2010; 29(6):1310–20.

4. Avants BB, Epstein CL, Grossman M, Gee JC. Symmetric diffeomorphic image registration with cross-correlation: Evaluating automated labeling of elderly and neurodegenerative brain. Medical Image Analysis. 2008; 12(1):26–41.

5. Zhang Y, Brady M, Smith S. Segmentation of brain MR images through a hidden Markov random field model and the expectation-maximization algorithm. IEEE Transactions on Medical Imaging. 2001; 20(1):45–57.

6. Reuter M, Rosas HD, Fischl B. Highly accurate inverse consistent registration: A robust approach. NeuroImage. 2010; 53(4):1181–96.

7. Dale AM, Fischl B, Sereno MI. Cortical Surface-Based Analysis: I. Segmentation and Surface Reconstruction. NeuroImage. 1999; 9(2):179–94.

8. Klein A, Ghosh SS, Bao FS, Giard J, Häme Y, Stavsky E, et al. Mindboggling morphometry of human brains. PLOS Computational Biology. 2017; 13(2):e1005350.

9. Wang S, Peterson DJ, Gatenby JC, Li W, Grabowski TJ, Madhyastha TM. Evaluation of Field Map and Nonlinear Registration Methods for Correction of Susceptibility Artifacts in Diffusion MRI. Front Neuroinform [Internet]. 2017 [cited 2020]; 11. Available from: https://www.frontiersin.org/articles/10.3389/fninf.2017.00017/full

10. Huntenburg JM. Evaluating nonlinear coregistration of BOLD EPI and T1w images [Internet]. Freie Universität Berlin; 2014 [cited 2020]. Available from: https://pure.mpg.de/pubman/faces/ViewItemOverviewPage.jsp?itemId=item_2327525

11. Treiber JM, White NS, Steed TC, Bartsch H, Holland D, Farid N, et al. Characterization and Correction of Geometric Distortions in 814 Diffusion Weighted Images. PLOS ONE. 2016; 11(3):e0152472.

12. Jenkinson M, Bannister P, Brady M, Smith S. Improved Optimization for the Robust and Accurate Linear Registration and Motion Correction of Brain Images. NeuroImage. 2002; 17(2):825–41.

13. Greve DN, Fischl B. Accurate and robust brain image alignment using boundary-based registration. NeuroImage. 2009; 48(1):63–72.

14. Lanczos C. Evaluation of Noisy Data. Journal of the Society for Industrial and Applied Mathematics Series B Numerical Analysis. 1964; 1(1):76–85.

**Supplementary Materials 2:**

See full paired t-test, and p-values for brain maps in Figure 1B below:

Clustering Coefficient:

Anterior Insula (pre-ASM < post- ASM): *t*(27) = -3.26, p = 0.002.

Superior Temporal Lobe (pre-ASM < post- ASM): *t*(27) = -3.18, p = 0.002.

Superior Frontal Lobe, pre-ASM < post- ASM): *t*(27) = -2.99, p = 0.004.

Path Length:

Anterior Insula (pre-ASM > post- ASM): *t*(27) = 3.15, p = 0.002.

Superior Temporal Lobe (pre-ASM > post- ASM): *t*(27) = 3.09, p = 0.002.

Superior Frontal Lobe (pre-ASM > post- ASM): *t*(27) = 2.87, p = 0.005.

Betweenness Centrality:

Superior Frontal Lobe (pre-ASM < post- ASM): *t*(27) = -2.89, p = 0.005.

Inferior Frontal Lobe (pre-ASM < post- ASM): *t*(27) = -2.97, p = 0.004.

Inferior Parietal Lobe (pre-ASM < post- ASM): *t*(27) = -3.07, p = 0.002.

**Supplementary Figure 1:**

**
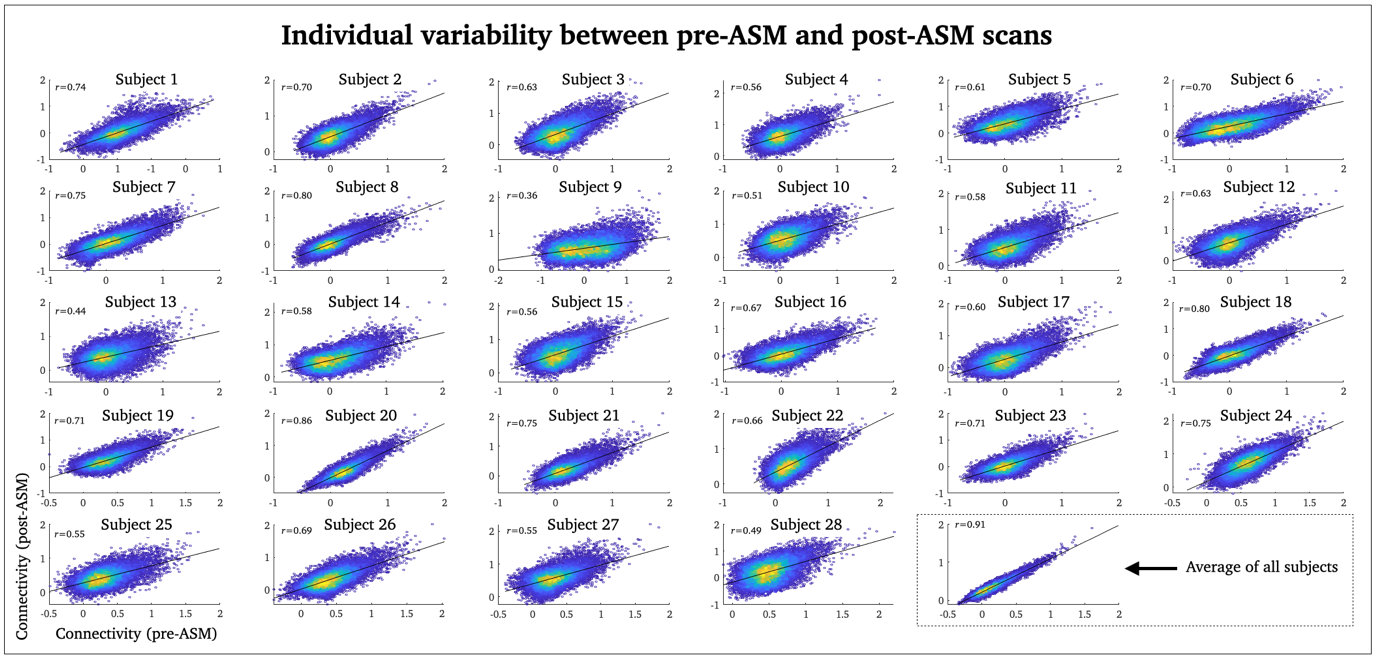
**

***Supplementary Figure 1:*** *In these scatterplots, the data points represent edge-wise Fisher’s z-scored correlation values from each patient’s 180 x 180 connectivity matrix. The first scan (pre-ASM) is on the x-axis, and the second scan (post-ASM) is on the y-axis. A Pearson correlation range between pre- and post-ASM from r = 0.36 to r = 0.86 on an individual level. After averaging the fMRI connectivity edge values across all subjects, Pearson’s r was 0.91. This finding suggests a good reproducibility between fMRI scans across subjects.*

**Supplementary Figure 2:**


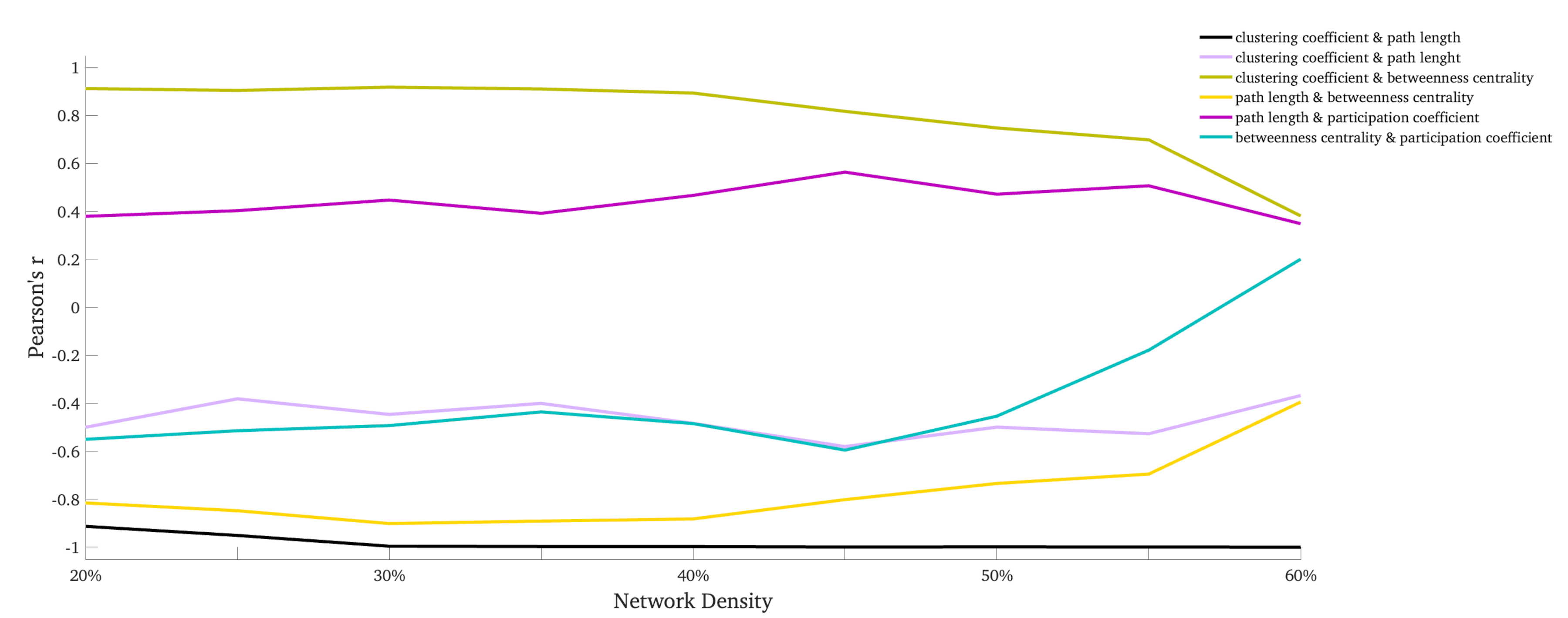


***Supplementary Figure 2:*** *Similarity between graph metrics used in this study, averaged across subjects and scans. Here we used a Pearson’s correlation coefficient (y-axis) to estimate the relationship between graph metrics across network densities (x-axis), averaging all nodes in the network.*

**Supplementary Figure 3:**


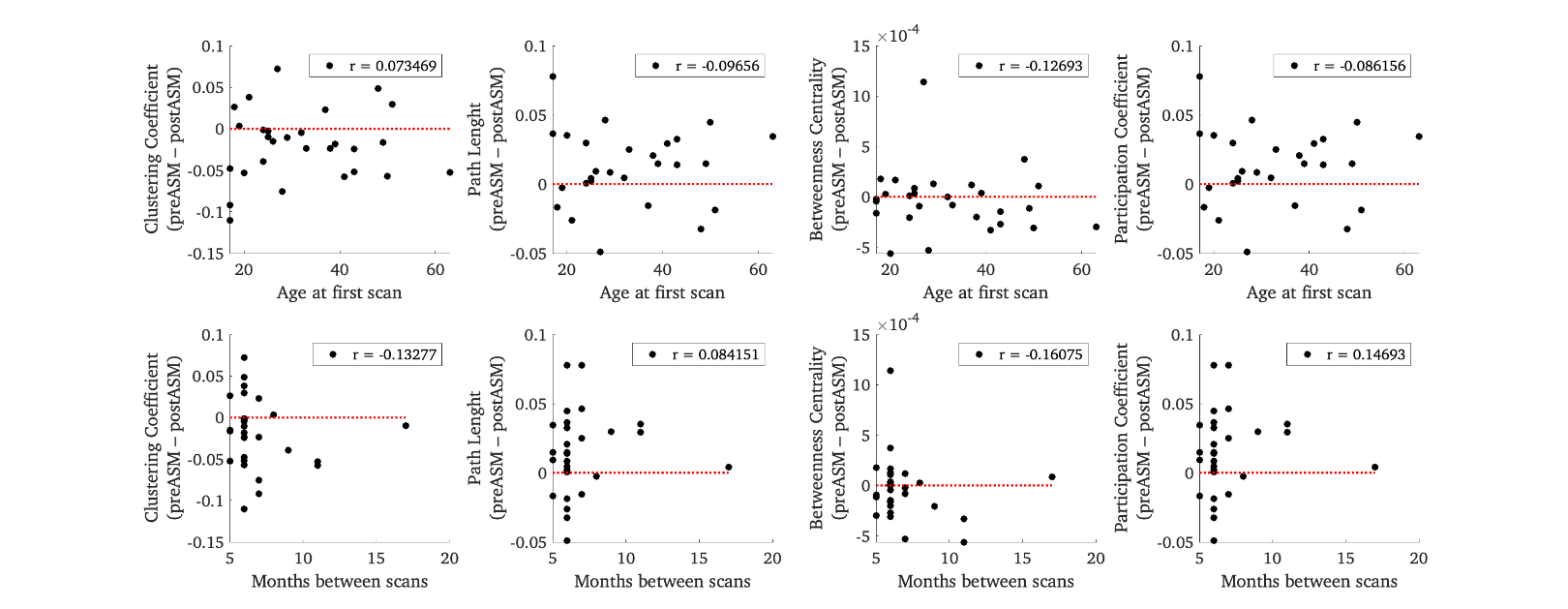


***Supplementary Figure 3:*** *Scatter plots and Pearson’s correlation values between differences in network metrics between scans (pre-ASM and post-ASM), and people’s age at the first scan (top row) and months between the pre-ASM and post-ASM scan (bottom row). No correlation was statistically significant at p < 0.05. Each dot represents a single subject’s whole brain average (average of all nodes) between pre- and post-ASM scans. We also conducted 4 linear regression models (difference between pre-ASM and post-ASM for the 4 network metrics), with age and months between scans as covariates. No significant regressions were seen for the clustering coefficient (R^2^ = .02, F(2, 25) = 0.26, p = .772), path length (R^2^ = .01, F(2, 25) = 0.15, p = .863), betweenness centrality (R^2^ = .10, F(2, 25) = 1.47, p = .249) or participation coefficient (R^2^ = .03, F(2, 25) = 0.35, p = .725).*
